# AI aided workflow for hip dysplasia screening using ultrasound in primary care clinics

**DOI:** 10.1038/s41598-023-35603-9

**Published:** 2023-06-07

**Authors:** Jacob L. Jaremko, Abhilash Hareendranathan, Seyed Ehsan Seyed Bolouri, Rod Fitzsimmons Frey, Sukhdeep Dulai, Allan L. Bailey

**Affiliations:** 1 Department of Radiology & Diagnostic Imaging, University of Alberta, Edmonton, Canada; 2 Exo Inc, Santa Clara, USA; 3 Sonance AI Inc, Edmonton, Canada; 4 Department of Surgery, University of Alberta, Edmonton, Canada; 5 Department of Family Medicine, University of Alberta, Edmonton, Canada

**Keywords:** Musculoskeletal system, Computational science

## Abstract

Developmental dysplasia of the hip (DDH) is a common cause of premature osteoarthritis. This osteoarthritis can be prevented if DDH is detected by ultrasound and treated in infancy, but universal DDH screening is generally not cost-effective due to the need for experts to perform the scans. The purpose of our study was to evaluate the feasibility of having non-expert primary care clinic staff perform DDH ultrasound using handheld ultrasound with artificial intelligence (AI) decision support. We performed an implementation study evaluating the FDA-cleared MEDO-Hip AI app interpreting cine-sweep images obtained from handheld Philips Lumify probe to detect DDH. Initial scans were done by nurses or family physicians in 3 primary care clinics, trained by video, powerpoint slides and brief in-person. When the AI app recommended follow-up (FU), we first performed internal FU by a sonographer using the AI app; cases still considered abnormal by AI were referred to pediatric orthopedic clinic for assessment. We performed 369 scans in 306 infants. Internal FU rates were initially 40% for nurses and 20% for physicians, declining steeply to 14% after ~ 60 cases/site: 4% technical failure, 8% normal at sonographer FU using AI, and 2% confirmed DDH. Of 6 infants referred to pediatric orthopedic clinic, all were treated for DDH (100% specificity); 4 had no risk factors and may not have otherwise been identified. Real-time AI decision support and a simplified portable ultrasound protocol enabled lightly trained primary care clinic staff to perform hip dysplasia screening with FU and case detection rates similar to costly formal ultrasound screening, where the US scan is performed by a sonographer and interpreted by a radiologist/orthopedic surgeon. This highlights the potential utility of AI-supported portable ultrasound in primary care.

## Introduction

Developmental dysplasia of the hip (DDH) leads to premature hip arthritis^1,2^. Screening is desirable because DDH is relatively common (incidence 1–3% of infants)^2^, it can be detected using a noninvasive tool (ultrasound, US), and effective treatment exists (Pavlik abduction harness)^3,4^. A study on cost-effectiveness of universal ultrasound screening in Austria showed screening was associated with a reduction in the number of non-surgical and surgical interventions^5^. A more recent study on Indian populations identifies logistic and financial challenges as key challenges in implementing universal screening^6^. Unfortunately, a conventional ultrasound performed by an expert sonographer and interpreted by an expert radiologist or surgeon is too costly and/or inaccessible for universal screening in most health-care systems worldwide. Portable US performed by lightly trained primary care providers at point-of-care, with decision support from artificial intelligence (AI), could potentially alter these economics and allow broad population-based infant screening.

In this study we evaluated the feasibility of using an FDA-cleared AI software for DDH detection (MEDO Hip) to screen for DDH in primary-care settings. We hypothesized that US/AI DDH screening could be integrated into busy clinical practices with case detection and recall rates similar to formal ultrasound screening where scanning is performed by a sonographer and interpreted by a radiologist/orthopedic surgeon.

## Methods

### Hardware and software

Hip US was performed with Philips Lumify handheld 12 MHz linear probes (Koninklijke Philips N.V., Amsterdam) connected by USB-C to Samsung S7 tablets (Samsung Electronics, Suwon-Si, South Korea), running the MEDO-Hip app (MEDO.ai, Edmonton, Canada, 2021) on the Android operating system. MEDO-Hip uses a UNet-like CNN model to identify structures like the acetabulum and femoral head that are important for DDH diagnosis, and automated geometric analysis with results combined in heuristics based on Graf methodology^7^ to provide clinical decision support. As is common with ‘beta’ software, several upgrades to the non-AI components of the MEDO Hip app were added during the course of this pilot study to improve user experience.

#### U-Net like CNN model

**Figure 1:**
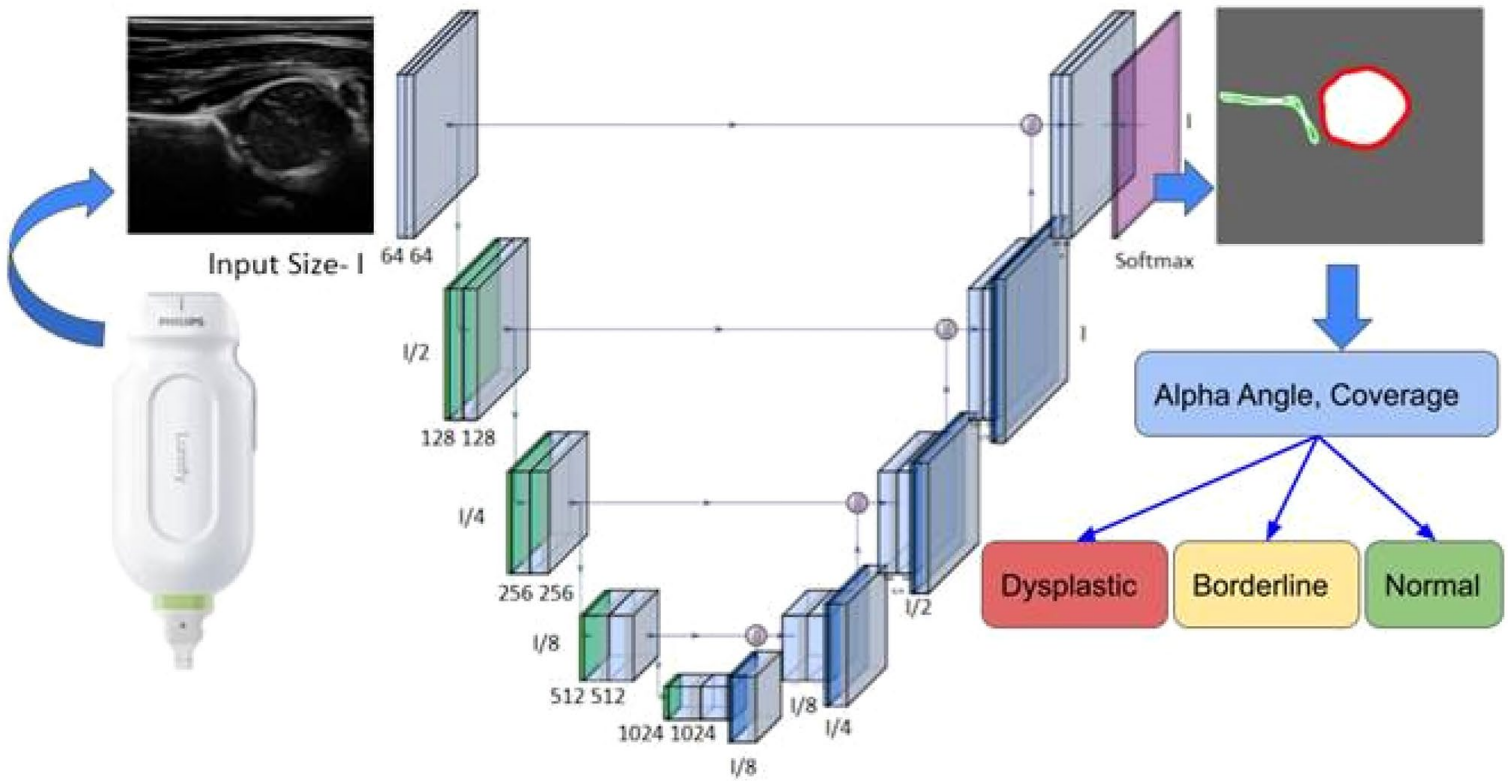
Schematic diagram of the U-Net-like CNN model used in MEDO-Hip. The CNN takes ultrasound images as input and returns a segmented image identifying key anatomic landmarks. Geometric measurements from segmented images are used to generate decision support/management advice.

A schematic representation of the model used is shown in Fig. 1. For training, individual ultrasound frames were provided as model inputs, normalized based on the overall average pixel intensity and resized to 256 × 256 pixels. Following Xavier random initialization the model was trained to minimize a DICE loss calculated between the predicted output mask and the ground truth annotation provided by human experts. The model providing the highest DICE score on the validation set was used for MEDO-Hip inference. Popular Python libraries including PyTorch and OpenCV were used for implementing the model and for resizing the images. Network output segmented anatomic landmarks are then further processed by automated geometric analysis measuring indices including the acetabular alpha angle and femoral head coverage, which are combined in heuristics based on Graf methodology to suggest management: “Healthy”, “FU Recommended” or “Inconclusive, Repeat Scan”.

#### Training data

The CNN model was previously trained on a large data set of labelled hip ultrasound images (10,572 femoral head and 16,657 acetabulum) obtained prospectively from 4 centers: University of Alberta Hospital (UAH) Edmonton (~ 70% of images), RJAH London (~ 10% of images), Royal Children's Hospital (RCH) Melbourne (~ 10% of images) and Westview Clinic prior to this study (~ 10% of images). With this large training dataset we did not perform data augmentation. The images were labeled by a team of sonographers, students and research assistants trained in hip ultrasound labeling by the lead radiologist (JJ). Annotations were performed using the ITK-Snap labeling software and MEDO’s annotation tool. Data were partitioned into a training set (80%) and a validation set (20%) that was disjoint at image, study, and patient levels.

This network architecture and training data were used to develop the MEDO Hip app for US-FDA clearance prior to this study. The current implementation study evaluated the combination of MEDO Hip software and Philips Lumify hardware as off-the-shelf products for clinical use. As is common with ‘beta’ software, several upgrades to the non-AI components of the MEDO Hip app were added during the course of this study to improve user experience.

### Scan/AI procedure

**Figure 2:**
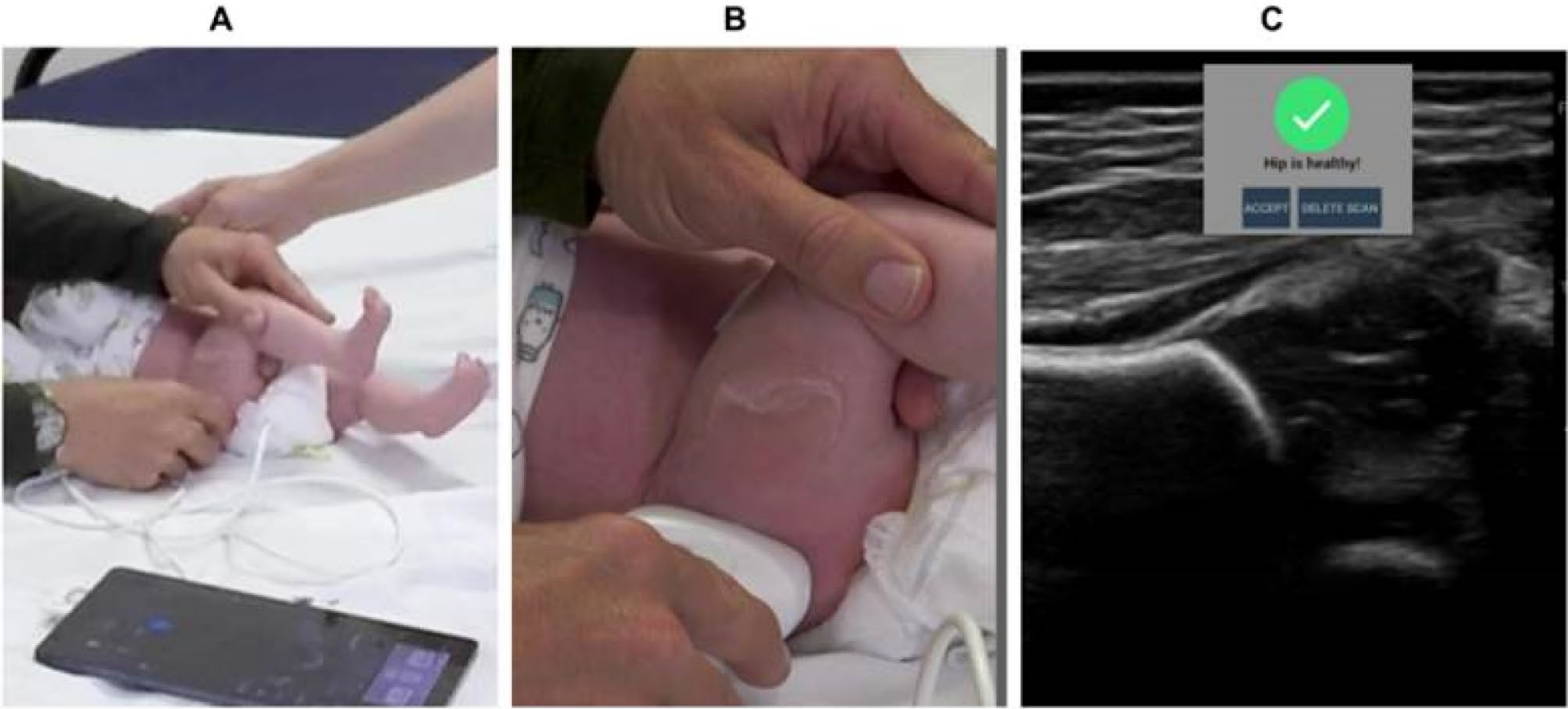
Scanning an infant’s right hip using handheld portable ultrasound probe and tablet (**A** and **B**), with Lumify and MEDO apps running (hip ultrasound image and app notification shown in **C**).

Once user credentials and patient identifiers are entered into MEDO Hip, scanning is initiated using the Lumify app (Fig. 2A). Following the approach outlined in video at https://youtu.be/rX1uVFHcqw8, the user ‘sweeps’ the US probe over each hip in the coronal plane(Fig. 2B), aiming to approximate the traditional Graf standard-plane image as closely as possible (Fig. 2C). An on-tablet real-time AI algorithm in MEDO Hip automatically detects and captures images containing anatomic landmarks as the user sweeps the probe across the hip. Once enough images suitable for AI analysis have been captured, the operator is notified the scan is complete. The captured images are uploaded to a cloud-based AI which performs more detailed processing, using a simplified technique intended to acquire images as close as practical to the Graf standard plane, to classify the hip as "healthy", "follow-up recommended" or "suboptimal (repeat scan)." Results return to the tablet (typically in < 1 min) as a notification, and a PDF report is generated for the medical record. Images can also be reviewed remotely on a web-based component of the app.

#### Clinical sites

We performed scans in 3 fee-for-service group-practice clinics in towns within 150 km of our referral city, in partnership with a Physician Collaborative serving a population ~ 80,000 via Center 1 (6 physicians) and Center 2 (14 physicians), and a Family & Walk-In Clinic serving a population ~ 100,000 via Center 3, 16 physicians. As an incentive, all clinics were given the ultrasound hardware to keep for further use after the study. At Center 1, we trained clinic registered nurses (RN) to perform scans, at Center 2, licensed practical nurses (LPN), and at Center 3, physicians. After users reviewed a training video and explanatory slide decks and our sonographer and radiologist demonstrated hip scanning, they were given increasing responsibility to perform scans themselves, initially under direct supervision over 2–4 scanning days, then unsupervised.

#### Scan protocol

This study was performed with Health Research Ethics Board approval (Pro00032107). Ultrasound scanning was performed in accordance with the relevant guidelines and regulations. At all clinics, hip screening was performed at already-scheduled infant wellness-check visits. As per ACR guidelines^8^, we sought to scan all infants presenting to the clinic at least once between 4 and 16 weeks of age, preferring to scan younger infants as these scans are technically easier to perform. Patients presenting outside the 4–16 week range in which scanning was technically feasible were also included. With parental informed consent and after obtaining basic demographic information including risk factors for DDH (3–5 min) we performed hip US (3–5 min), received automated AI results (1 min), and provided these to the parents and the attending physician.

At all 3 sites we used an "internal follow-up" protocol to facilitate user training and ensure only high-quality external DDH referrals. Any baby with a hip flagged as 'follow up' on a scan would return to the same clinic, preferably on a day when a study sonographer was present. On those days the clinic user and, ideally, our sonographer, each performed two scans of each hip. In practice, due to scheduling constraints a sonographer was frequently not available; in this case only the clinic user would perform the repeated scan. If a hip still could not be confirmed “normal” on these follow-up scans, the infant was referred to a pediatric orthopedic clinic.

#### Evaluation

We assessed the number of users trained and babies scanned at each site, technical failure rates and screening recall rate across users, sites, and study dates.

## Results

### Number of scans

We performed 369 scans Feb 1, 2021–Mar 31, 2022, across sites (Center 1: 176, Center 2: 70; Center 3: 60), in 306 unique patients, mean age 45 days (range 3–193 days), 156 (51%) female, 26 breech deliveries (9%), 29 (9%) with positive family history of DDH, and 32 (10%) Indigenous. Our ultrasound users included 2 registered nurses (RN) (158 infants), 4 licensed practical nurses (LPN) (49 infants), 3 assisting sonographers (24 infants), and 4 physicians (65 infants). The user performing the scan was not recorded in 10 infants.

### Scan results

As seen in Table 1, 6% of infants could not be scanned at all, 80% were normal at first scan, 12% were normal at internal follow-up scan (triggered by app technical failure in 4% and by AI interpretation in 8%), and six infants (2%) were referred to orthopedic clinic, all ultimately treated for DDH. Of the 6 infants treated for DDH (5 female), 1 was Indigenous, 1 was a breech infant with positive family history, and the other 4/6 had no risk factors for DDH.

**Table 1:** Scan results and management during the pilot study.

Scan results and AI interpretation	N (%)	Management	Comments
Could not scan at all	18 (5.9%)	No further imaging	Typically, large or uncooperative infants near upper age limits; unsuitable for screening
Normal	244 (80%)	No further imaging	
Suboptimal	12 (3.9%)	Internal FU	Poor scan technique: all were normal in FU
Follow up	32 (10%)	Internal FU: * 26 (8.2%) normal * 6 (2.0%) dysplastic	Six infants detected and treated for DDH (5 Pavlik harness, one closed reduction)

### Follow-up rates

**Figure 3:**
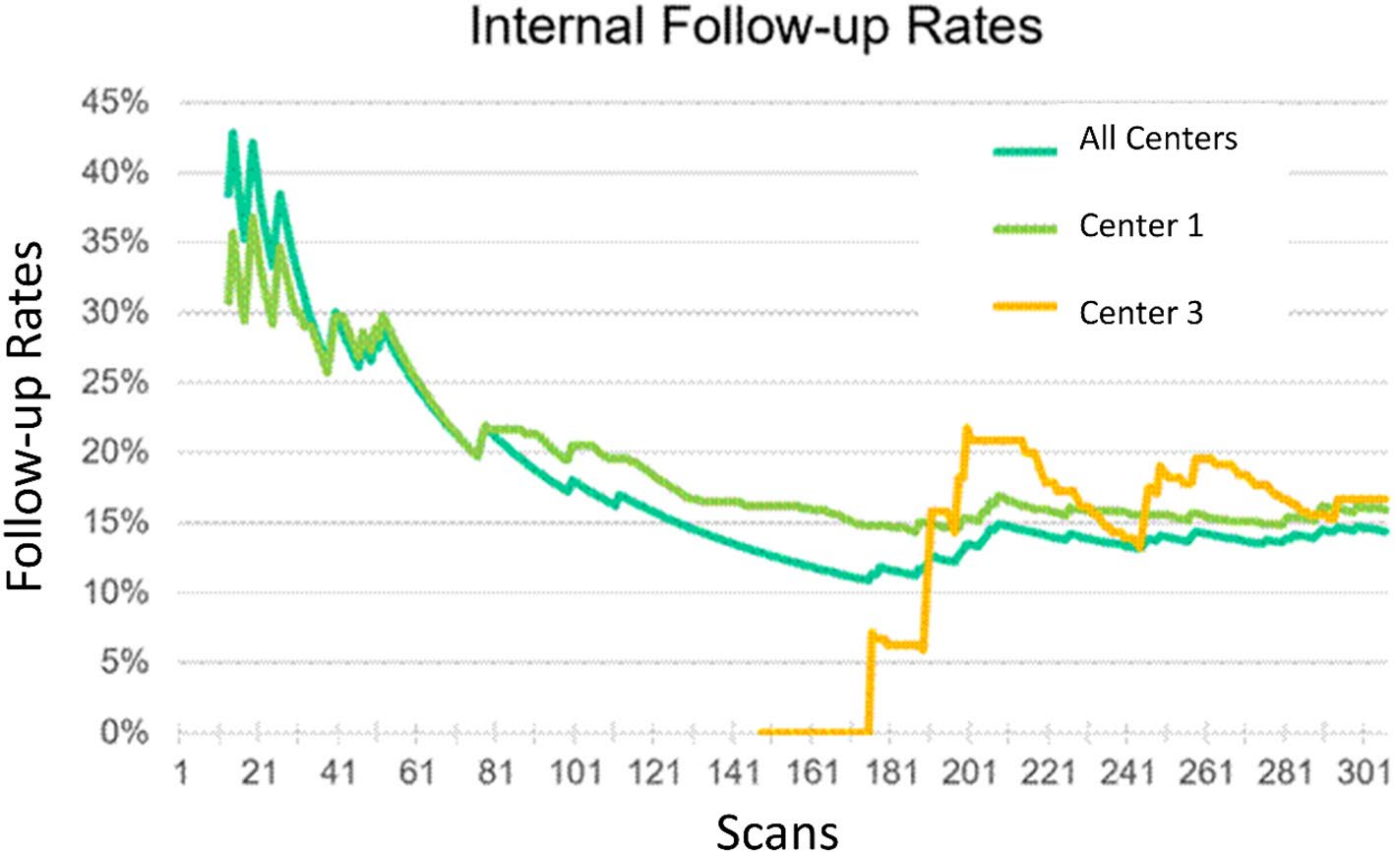
FU rates as a proportion of total initial scans performed, across all sites, at Center 1 (Westland), and at Center 3(Red Deer added as a site halfway through the study).

FU rates decreased substantially as users gained experience. The proportion of scans requiring internal FU (due to "suboptimal" scan or AI "follow-up" recommendation) decreased from 30 to 40% for nurses at Sites 1/2 and 20% for scans by physicians at Site 3, to a steady-state near 14% after ~ 60 scans/site (Fig. 3).

## Discussion

We found that DDH screening at primary-care clinics, performed by clinic staff using automated AI diagnosis from images obtained by handheld portable ultrasound, was feasible when integrated with routine well-baby visits, whether performed by RN, LPN or physicians. Our recall rate to internal FU and our DDH treatment rate, 14% and 2% respectively, are similar to the 17% and 3% observed in the 'general screening' arm in a well-known Norwegian study of ~ 12,000 neonates using conventional DDH ultrasound performed by expert physician^9^. A UK study of 48,000 neonates using conventional DDH ultrasound performed by expert sonographers had lower rates for FU (6.6%) and treatment (0.3%)^1^. If we exclude app technical difficulties (inevitable in 'beta' software), our FU rate was 10%, intermediate between these studies. Our DDH treatment rate of 2% closely matches the expected DDH incidence^10^. Crucially, we note that 4/6 infants we detected with DDH had no risk factors and may not have been detected at all without our screening program.

Screening program cost-effectiveness is closely linked to FU rates. Our high initial FU rate at each site justified initial onsite sonographer support during 'onboarding'. FU rates stabilized after ~ 60 infants/site at ~ 14%: 4% due to app technical errors (requiring ongoing beta software refinement), and 10% due to AI "follow-up" recommendation. At internal follow-up by study sonographer or experienced primary care user with the AI app, 80% of cases were normal and the other 20% (2% of all cases, 6 infants) were all later confirmed to have DDH after external referral. Initial FU rates were substantially lower (20% vs. 40%) when physicians performed scans vs. nurses. These findings demonstrate that hip ultrasound remains difficult to perform, and that even with our simplified protocol and AI decision support, selected cases will still need expert assessment. We found that internal follow-up was a necessary component of the screening program, but note that this should represent a much smaller cost to the health-care system and less inconvenience to families than an external tertiary referral.

The main practical challenge in hip ultrasound is to train sonographers to acquire scans that capture all the clinically important anatomical landmarks. Novice users find it hard to recognize when they have obtained a good image. Our AI tool addresses this issue by providing confirmation to the user when a sufficient number of high-quality images have been acquired. Given the difficulty of this ultrasound examination, we feel the primary role of AI is to enhance diagnostic confidence, as non-expert users may be hesitant to proceed without it.

Our study had limitations. This was a small pilot study. Diagnostic performance could only be assessed on a limited basis, because it was not feasible to also perform gold-standard conventional ultrasound on all babies. The underlying image analysis approach was previously studied retrospectively in > 1200 hips with very high diagnostic accuracy in tertiary-hospital settings^3^, which may not generalize to our real-world setting. Our results are for the combination of MEDO-Hip software and Philips Lumify hardware, and may not apply directly to other ultrasound hardware, which may have different image quality. Our results are obtained in a suburban population with a relatively high Indigenous component; incidence of DDH does differ in other populations.

The performance of AI algorithms can be affected by image quality. In our earlier study, without AI based quality assessment^4^ we found that there were occasional unusual/outlier AI results in poor image quality images. The tool reported here only accepts scans that contain all necessary clinical landmarks which ensures adequate image quality.

Overall, we found that an AI app evaluating cine-sweep hip ultrasound enabled primary-care clinic staff with only brief training to routinely perform DDH screening with recall and detection rates similar to conventional ultrasound applied by highly-trained expert users in a tertiary-hospital setting. Integrating AI decision support with point-of-care ultrasound offers a practical approach to population screening for DDH that has the potential to shift the cost–benefit balance in favor of universal US screening and may be a model for other uses of ultrasound with AI decision support in primary care.

## Data Availability

The datasets analyzed during the current study are not publicly available due to our institutional guidelines on data sharing but are available from the corresponding author on reasonable request after signing a Data Transfer Agreement (DTA).
